# Attenuation of carbohydrate metabolism and lipid profile by methanolic extract of *Euphorbia helioscopia* and improvement of beta cell function in a type 2 diabetic rat model

**DOI:** 10.1186/s12906-022-03507-2

**Published:** 2022-01-25

**Authors:** Imtiaz Mustafa, Haseeb Anwar, Shahzad Irfan, Humaira Muzaffar, Muhammad Umar Ijaz

**Affiliations:** 1 Department of Physiology, Faculty of Life Sciences, Government College University, Faisalabad, Pakistan; 2 Department of Zoology, Wildlife and Fisheries, University of Agriculture, Faisalabad, Pakistan

**Keywords:** *Euphorbia helioscopia*, Antioxidant, Methanolic extract, Antidiabetic, Carbohydrate metabolism

## Abstract

**Background:**

Traditional plant-based remedies prescribed to treat diabetes have shown promise in research-based setting. Current research was conducted to examine the antidiabetic and antioxidant effects of methanolic extract of a folk herbal plant *Euphorbia helioscopia* in a rat model of type 2 diabetes.

**Methods:**

Diabetes was induced in male Wistar rats by administering 5% sucrose in drinking water and cafeteria diet for 8 weeks with subsequent nicotinamide and streptozotocin administration. Diabetic rats were then distributed into four individual groups (*n* = 8); Positive control (PC; no treatment), standard control (SC; Metformin @ 10 mg/kg bw), treatment 1 (EH1, *E. helioscopia* methanolic extract @200 mg/kg bw) and treatment 2 (EH2, *E. helioscopia* methanolic extract @400 mg/kg bw). After 21 days of treatments, the rats were decapitated for blood collection. Serum was evaluated for antidiabetic potential, antioxidant and lipid profile, thyroid hormone, amylin, leptin, and carbohydrate metabolic enzymes. Data were analyzed statistically by one-way analysis of variance (ANOVA).

**Results:**

Serum levels of glucagon, glucose and C-peptide were significantly (*P* ≤ 0.05) decreased in EH1 (1915.33 ± 98.26^a^ pg/ml, 122.59 ± 2.99^a^ mg/dl, 277.59 ± 28.41^a^ pg/ml respectively) and EH2 (1575.28 ± 56.46^a^ pg/ml, 106.04 ± 5.21^a^ mg/dl, 395.06 ± 42.55^a^ pg/ml respectively) as compared to the PC (3135.78 ± 189.46^b^pg/ml, 191.24 ± 17.75^b^mg/dl, 671.70 ± 109.75^b^ pg/ml respectively) group. A similar trend was observed in serum insulin levels in EH1 and EH2 groups. The plant’s methanolic extract effectively reduced the total oxidant status (TOS) and MDA levels in the diabetic rats and increased the total antioxidant capacity (TAC) along with an increased level of SOD, Catalase, Paraoxonase, and arylesterase. The plant extract also induced antihyperlipidemic activity and recovered the thyroid hormones, amylin, and leptin levels to normal. The activity of different carbohydrate metabolic enzymes like Pyruvate Kinase, Glucose 6 phosphate dehydrogenase, phosphofructokinase, and glucokinase has also been restored by the extract treatment.

**Conclusion:**

Current study indicates the antioxidant and antidiabetic potential of *E. helioscopia* methanolic extract in normalizing the lipid profile, thyroid hormones, amylin, leptin, and carbohydrate metabolism in type 2 diabetic rat model.

## Background

Diabetes mellitus, one of the most common metabolic conditions causing hyperglycemia due to disruption in the metabolism of carbohydrates, proteins, and lipids induced by a relative or absolute decline in insulin secretion and insulin action [1]. The incidence of diabetes in modern society is growing gradually and becoming an epidemic. There are currently 415 million diabetic patients globally and by 2040 it is estimated to be around 642 million [2]. Pakistan has 27.4 million diabetic patients of age ≥ 20 years estimated by the second national diabetes survey of Pakistan (NDSP-II 2016–17) at 26.3% of diabetes prevalence [3, 4].

The World Health Organization (WHO) reports that about three-quarters of the population, primarily in Latin American, African and Asian countries, relies on herbal preparations in their conventional primary health care medicine system [5]. Pakistan has large biodiversity of genetic plants and, owing to inadequate access to health assistance, medicinal plants have been widely used by almost all populations [6].

Natural products continue to represent the main source of therapeutic and preventive regimens of different illnesses. The family of Euphorbiaceae contains a variety of medicinal plants possessing a wide range of different reported therapeutic effects. *Euphorbia helioscopia* is an annual herbaceous medicinal plant belonging to the spurge family Euphorbiaceae. It contains 24 secondary metabolites, including euphornin, euphoheliosnoid D, euphornin (B, C), euphoscopin (A-C, F, J), epieuphorscopin (A, B), hemistepsin, helioscopinolide (B, C), 2 alpha hydroxy helioscopinolide B, 4 5-dihydroblumentol A, guaiane lactone, icariside aglycone B2, 2′ 4′ 4′-trihdroxy chalcone, echinatialicochalcone (A, B), galabrone and 4′ 5 7-trihdroxyflavanone [7]. These secondary metabolites induce numerous pharmacological effects including vasodepressor activity, anti-inflammatory, antimicrobial activity, antioxidant, anti-tumor, and wound healing activities [8–12]. Traditionally, the plant has been used to treat a variety of pathological disorders, including skin diseases, warts, bowel parasites, migraines, and gonorrhea [13]. The medicinal plants having antioxidant and anti-inflammatory activities can reduce hyperglycemia [14]. We previously reported the in vitro antioxidant and antidiabetic activities of *E. helioscopia* extracts in which the methanolic extract showed maximum antidiabetic and antioxidant activity as compared to ethanolic and aqueous extracts of the plant [15]. So the present study was conducted to examine the ability of methanolic extract of *E. helioscopia* to modulate oxidative stress, hyperglycemia, and carbohydrate metabolism in a type 2 diabetic rat model.

## Methods

### Procurement of plant

The plant *Euphorbia helioscopia* was locally collected from the premises of Government College University, Faisalabad, Pakistan, from October 15, 2020, to October 30, 2020. The plant was identified by the expert Botanist (Dr. Qasim Ali) from the Department of Botany, Government College University Faisalabad, Pakistan, with a specimen voucher number, 247-bot-2020.

### Extract preparation

After washing with distilled water, the plant was shade dried and grounded into a fine powder form. Two hundreds and fifty grams of this powder was soaked for 72 h in 1250 ml of methanol with periodic stirring and mixing. The solution was subsequently filtered through Whatman® filter paper. The process was repeated thrice, and the final filtrates were concentrated in a rotary evaporator at 40 °C and finally transferred into labeled petri dishes. The petri dishes were kept in an incubator at 40 °C until dried properly. The yield of the extract was 15.7% of the dry weight of the plant powder. Thereafter the extract was stored at 4 °C until further use.

### Experimental animals and induction of diabetes

After the approval from Animal Care and Ethical Committee, Government College University Faisalabad (GCUF/ERC/2223), 48 male albino (Wistar strain) rats of age 2 ± 1 week, weighing 60 ± 10 g were procured from the animal experimental station of Department of Physiology, Government College University, Faisalabad, Pakistan. The rats were kept in wired cages with ad libitum approach to cafeteria diet and 5% sucrose in drinking water at standard conditions (temp = 26 °C ± 2 °C; light = 12 h light and dark cycle; ambient humidity = 40–60%). Another group of eight rats as negative control (*n* = 8; NC) were kept in the same conditions except for 5% sucrose in drinking water and cafeteria diet, during the complete duration of the experimental trial. The cafeteria diet contained cheese, peanuts, various biscuits, potato chips, corn chips, crackers, pizza, and various chocolates, along with a control chow diet. Any five of these cafeteria diets were given to the rats daily in surplus amounts. The foods ration was changed daily to sustain the diversity by substituting three foods with new food items. So that the rats did not get the same diets for more than two successive days at one time [16]. The cafeteria diet and 5% sucrose in drinking water were given to the rats starting from the age of 2 + 1 weeks for 56 days (8 weeks). After 8 weeks the rats weighing 300 ± 20 g, with blood glucose level ≥ 150 mg/dl were fasted overnight and administered nicotinamide (NIC; 110 mg/kg bw; ip) in normal saline [17]. After 15 min of the administration of nicotinamide, the dose of freshly prepared streptozotocin (STZ; 65 mg/kg bw; ip) in cold citrate buffer (0.1 M, pH 4.5) was administered [17]. Hyperglycemia was confirmed after 72 h by measuring blood glucose levels using a glucometer (Glucocard 01-mini, Arkray Factory Inc., Japan) [17]. The 32 rats with blood glucose level ≥ 250 mg/dl were divided equally into the following 4 groups PC (Positive control; no treatment), SC (Standard control group; Treated with metformin 10 mg/kg bw [18] via oral lavage), EH1 (Treatment group-I fed on CMD and plant extract dose 200 mg/Kg BW via oral lavage), EH2 (Treatment group-II fed on CMD and plant extract dose 400 mg/Kg BW via oral lavage). The doses of the plant extract were followed by previous studies [19–21] on the antidiabetic potential of the methanolic extract of plants from the euphorbeacea family. Suspensions of metformin in normal saline and plant extracts in distilled water were made on daily basis. Volumes of these suspensions were calculated according to the bodyweight of each rat in the relevant group. The calculated volume of the suspension was delivered to the rats via oral lavage using an intragastric tube. No motility or toxic effects were observed in any experimental group. After the completion of 21 days of treatment, the rats were sacrificed by cervical decapitation under ether anesthesia for the collection of blood directly from the jugular vein in vacutainers without anticoagulant. Serum was isolated at 2000 g centrifugation for 10 min and kept at − 20 °C till further analysis. Pancreas was excised and kept in 10% formalin for histopathology studies.

### Serum hormones levels and C-peptide

Commercially available ELISA kits manufactured by Elabscience, USA were used to measure the serum levels of various hormones like Triiodothyronine (fT_3_; ng/ml; Cat. No. E-EL-0079; Sensitivity: 0.937 pg/ml; Range of Detection: 1.6–100 pg/ml), Thyroxin (fT_4_; μg/dl; Cat. No. E-EL-0122; Sensitivity: 0.937 pg/ml; Range of Detection: 1.6–100 pg/ml), Thyroid Stimulating Hormone (TSH; ng/ml; Cat. No. E-EL-R0976; Sensitivity: 0.747 ng/ml; Range of Detection: 1.25–80 ng/mL), Insulin (Rat INS; ng/ml; Cat. No. E-EL-R2466; Sensitivity: 0.20 ng/ml; Range of Detection: 0.309–20 ng/ml;), C-peptide (Rat C-P; pg/ml; Cat No: E-EL-R3004; Sensitivity: 9.4 pg/mL; Range of Detection: 15.64-1000 pg/mL), Leptin (Rat LEP; ng/ml; Cat. No. E-EL-R0528; Sensitivity: 0.11 ng/ml; Range of Detection: 0.16–0.92 ng/ml), and Amylin (Rat IAPP; pg/ml; Cat. No. E-EL-R2448; Sensitivity: 37.51 pg/ml; Range of Detection: 62.51–4000 pg/ml). Coefficient of variation for all the kits was < 10%. The manuals provided in the kits were followed for the assay procedure for all the above mentioned hormones and C-peptide.

### Carbohydrate metabolic enzymes and superoxide dismutase

Serum levels of Carbohydrate metabolic Enzymes, Pyruvate Kinase (Rat PK; ng/ml Cat. No. E-EL-R0837), Phosphofructokinase (Rat 6.PFK; ng/ml; Cat. No. E-EL-R1214; Sensitivity: 0.957 ng/mL; Range of Detection: 1.6–100 ng/ml), Glucose 6-Phosphate Dehydrogenase (Rat G6PD; μlU/ml; Cat. No. E-EL-R0428; Sensitivity: 0.11 ng/ml; Range of Detection: 0.16–11 ng/ml), and glucokinase (Rat GCK; ng/ml; Catalog No. E-EL-R0426 Sensitivity: 0.39 ng/mL; Range of Detection: 0.63–41 ng/ml), and Superoxide dismutase (Rat SOD; Catalog No. E-EL-R1424; Sensitivity: 0.11 ng/ml; Range of Detection: 0.16–11 ng/ml) were analyzed by using ELISA kits manufactured by Elabscience, USA. The coefficient of variation for all the ELISA kits was < 10%. The manuals provided in the ELISA kits were followed for the assay procedure for all the above-mentioned carbohydrate metabolic enzymes and superoxide dismutase.

### Serum lipid profile and glucose level

Commercially available colorimetric assay kits manufactured by Sigma-Aldrich, Germany, were used for the serum levels of Total Cholesterol (Catalog Number CS0005; mg/dl; Detection range: 1–5 μg), Triglycerides (Catalog Number MAK266; mg/dl; Sensitivity: 2 pmole–10 nmole; Detection range: 2–10,000 μM range), HDL-Cholesterol (Catalog Number MAK045; mg/dl; Detection range: 1–5 μg). The manuals provided in the kits were followed for the assay procedure of serum total cholesterol, serum triglycerides, and serum HDL-cholesterol level determination. Serum LDL-Cholesterol was calculated by using the Friedrick equation. Serum glucose was quantified through Bioclin® Glucose Monoreagent diagnostic kit having detection range of 2-500 mg/dl with CV% < 3.11.

### Total antioxidant capacity (mmol Trolox_equiv._/L)

The method for evaluating total antioxidant capacity (TAC) in serum samples was previously defined by Nisar et al. [22]. In short, the antioxidants present in the sample bleach the ortho dianisidine color in the assay reagent. Increased antioxidant levels in the sample contribute to higher bleaching and reduced absorbance, suggesting an inverse standard curve. The Biolab® 310 semi-auto analyzer was used with biochromatic wavelength modification (660 and 870 nm) by calibrating Trolox standards at 1, 3, 5, and 7 mmol/L concentrations. This assay had a minimum observable value of 0.19 mmol/7 and linearity of up to 8 mmol Trolox_equivalent_/l, with a coefficient of variance < 3% in the intra-assay.

### Total oxidant status (TOS; μmol H_2_O_2 equiv._/L)

The serum TOS was measured by using the technique previously adopted by Nisar et al. [22]. The standard curve was constructed from different hydrogen peroxide (H_2_O_2_) concentrations and the TOS was represented as μmol H_2_O_2_ equivalent/l. The assay’s detection range was < 3% and the CV of the Intra assay was held < 10% and linearity was up to 250 μMol of H_2_O_2_ equivalent /l [22].

### Paraoxonase (PON1) activity (U/L)

For the estimation of PON1 activity, the method defined by Anwar et al. [23] was used. Using the reference formula, the activity of the enzyme was measured and stated in Unit/min/l. Intra assay CV was < 10% and the paraoxon hydrolysis rate was constant for up to 6 min. The minimum measurable activity was between 80 and 100 U/min/L for this assay.

### Arylesterase activity (KU/L)

By adopting the protocol as previously adopted by Anwar et al. [23], the activity of arylesterase was evaluated using the given equation in the reference procedure. The CV was < 7% and the initial hydrolysis rate was stable for up to 5 min.

### Serum malondialdehyde level (pM/dl)

The serum level of malondialdehyde (MDA) was calculated by the thiobarbituric reactive substance assay (TBARS) reported by Al-Assaff and Takruri [24], which is based on the pink pigment-producing MDA reaction with thiobarbituric acid (TBA). For this, 500 μl of the serum was mixed with 2.5 ml of 10% TBA solution, then the tube was put for 15 min in a boiling water bath. It was then cooled in cold water and centrifuged for 5 min at 3000 g. In a test tube, 2 ml of the supernatant was applied to 1 ml of 0.67% TBA solution and put for another 15 min in a boiling water bath, and finally cooled in tap water. The absorbance was measured at 532 nm using Biolab 310, Biolab Scientific Ltd. Canada against distilled water as a blank [24, 25].

### Catalase activity assay (KU/min)

Catalase enzyme activity was measured by mixing the 100 μl of serum sample in 1000 μl substrate (649 mmol/ml H_2_O_2_ in 59 mmol/l phosphate buffer saline, pH 7.4). In the control test tube, the substrate was replaced with 1 ml distilled water and in the standard tube, the serum was replaced with 100 μl of distilled water. After 3 min of incubation at 37 °C, the reaction was terminated with ammonium molybdate (32.4 mM). The optical density of the yellow-colored complex produced from molybdate and H_2_O_2_ was calculated at 374 nm against the blank using Biolab 310, Biolab Scientific Ltd. Canada. The equation of first-order reaction (k) was used to measure catalase enzyme activity:

$$kU=\frac{2.303}{t}\times \left[\mathit{\log}\frac{S{}^{\circ}}{S-M}\right]\times \frac{\mathrm{Vt}}{\mathrm{Vs}}$$

S°: optical density of standard; S: optical density of test sample; M: optical density of control sample; V_t_: overall volume of all components in the test tube; V_s_: total volume of serum sample [26].

### Histopathology of pancreas

Pancreas tissues were taken out from the 10% formalin and embedded in paraffin to make the blocks. Sections of 4 to 5 μ were sliced with Microtome [Bk-Mt268m; Biobase Biodustry (Shandong) Co., Ltd.] for histopathological examination. The tissue sections were placed on a glass slide coated with albumin. It was then deparaffinized, rehydrated, and finally stained with hematoxylin and eosin stain. The stained slides were then examined under a light microscope after DPX mounting and covered with a coverslip [27].

### Statistical analysis

The SPSS software version 23 was used for statistical analysis. All datasets were statistically demonstrated as mean ± standard error. One-way Analysis of Variance (ANOVA) was used to find the significance of difference among various groups followed by turkey’s post hoc test. The level of significance was actively considered at *p* < 0.05 with 8 number of samples in each group (*n* = 8).

## Results

### Antioxidant activity

#### Effect of *Euphorbia helioscopia* on oxidative stress

The mean serum TAC level was increased significantly (*P* ≤ 0.05) in the EH2 group (2.5 ± 0.47^b^ mM/l) as compared with other experiment groups (Table 1). There was also a non-significant increase in TAC value in EH1 (1.61 ± 0.2^ab^ mM/l) and SC group (1.4 ± 0.17^ab^ mM/l) compared with the PC group (1.04 ± 0.41^a^ mM/l). The difference between EH1 and EH2 was non-significant. *Euphorbia helioscopia* extract significantly (*P* ≤ 0.05) decreased the TOS in the EH1 (18.94 ± 0.82^a^ uM/l) and EH2 groups (12.97 ± 0.61^a^ uM/l) (Table 1) as compared with the PC (31.13 ± 3.34^b^ uM/l) group but the difference between the mean TOS value of EH1 and EH2 was not significant. The mean TOS value in the SC group was also decreased as compared with the PC group but the change was not significant. *Euphorbia helioscopia* methanolic extract significantly (*P* ≤ 0.05) increased the antioxidant enzyme SOD concentration in serum at the higher dose of 400 mg/kg in the EH2 group (8.39 ± 1.06^b^ ng/ml) as compared to the PC group (5.17 ± 0.8^a^ ng/ml) but the increase in the Mean SOD value in SC and EH1 group was not significant as compared with the PC group (Table 1). A dose-dependent increase in serum catalase activity in the EH1 (19.37 ± 4.29^b^ KU/min) and EH2 groups (31.18 ± 1.78^c^ KU/min) as compared to the PC group (10.07 ± 0.71^a^ KU/min) was observed. There was also a significant increase in the catalase activity of serum catalase activity of the SC group (17.56 ± 0.68^b^ KU/min) as compared with the PC group (10.07 ± 0.71^a^ KU/min). Statistically significant (*P* ≤ 0.05) decrease was observed in MDA levels in the EH2 group (0.82 ± 0.5^a^ pM/dl) group as compared to the PC group (2.64 ± 0.47^bc^ pM/dl). The lower dose of the plant non significantly decreased the serum MDA levels in the EH1 group (1.17 ± 0.28^bc^ pM/dl) and SC group (1.06 ± 0.21^ab^ pM/l) as compared to the PC group (2.64 ± 0.47^bc^ pM/dl). There was a significantly (*P* ≤ 0.05) increase in the serum arylesterase and paraoxonase activities in the EH2 group (163.41 ± 7.77^b^ KU/l and 31.28 ± 3.58^b^ U/l respectively) as compared to the PC group (125.71 ± 1.78^a^ KU/l and 17.18 ± 0.98^a^ U/l respectively). Activities of these enzymes were also increased in the EH1, and SC groups, but the change was non-significant as compared with the PC group (Table 1).

**Table 1:** Effect of *Euphorbia helioscopia* on oxidative stress markers

Groups	PON1 (U/L) ***p*** = 0.005	ARY (KU/L) ***p*** = 0.006	TAC (mM/L) ***p*** = 0.036	TOS (uM/L) ***p*** = 0.001	MDA (pM/dl) ***p*** = 0.02	Catalase (KU/min) ***p*** = 0.0	SOD (ng/ml) ***p*** = 0.041
**NC**	18.60 ± 3.62^a^	140.88 ± 5.5^ab^	1.17 ± 0.2^ab^	22.56 ± 4.72^ab^	1.66 ± 0.34^ab^	16.93 ± 2.06^ab^	7.83 ± 0.16^ab^
**PC**	17.18 ± 0.98^a^	125.71 ± 1.78^a^	1.04 ± 0.41^a^	31.13 ± 3.34^b^	2.64 ± 0.47^bc^	10.07 ± 0.71^a^	5.17 ± 0.8^a^
**SC**	24.59 ± 0.53^ab^	147.18 ± 6.83^ab^	1.4 ± 0.17^ab^	23.26 ± 1.97^ab^	1.06 ± 0.21^ab^	17.56 ± 0.68^b^	6.52 ± 0.89^ab^
**EH1**	26.24 ± 2.57^ab^	145.29 ± 6.31^ab^	1.61 ± 0.2^ab^	18.94 ± 0.82^a^	1.17 ± 0.28^bc^	19.37 ± 4.29^b^	7.87 ± 0.15^ab^
**EH 2**	31.28 ± 3.58^b^	163.41 ± 7.77^b^	2.5 ± 0.47^b^	12.97 ± 0.61^a^	0.82 ± 0.5^a^	31.18 ± 1.78^c^	8.39 ± 1.06^b^

Data are expressed as means ± standard errors of a total of 8 samples for each group. Values with different letters in a column are significantly different (*P* ≤ 0.05). NC, negative control group; PC, positive control group (Diabetic rats without any treatment); SC (Standard control group (Diabetic rats treated with metformin at 1 mg/kg body weight via oral lavage); EH1, Treatment group-I (Diabetic rats given methanolic extract of *Euphorbia helioscopia* at dose of 200 mg/Kg BW via oral lavage); EH2, Treatment group-II (Diabetic rats given methanolic extract of *Euphorbia helioscopia* at dose of 400 mg/Kg BW via oral lavage); PON1, Paraoxonase activity; ARY, Arylesterase activity, TAC; Total antioxidant capacity, TOS; Total oxidant status, SOD; Superoxide dismutase

### Antidiabetic activity

#### Effect of *Euphorbia helioscopia* on insulin, glucagon, glucose, and C-peptide

**Fig. 1:**
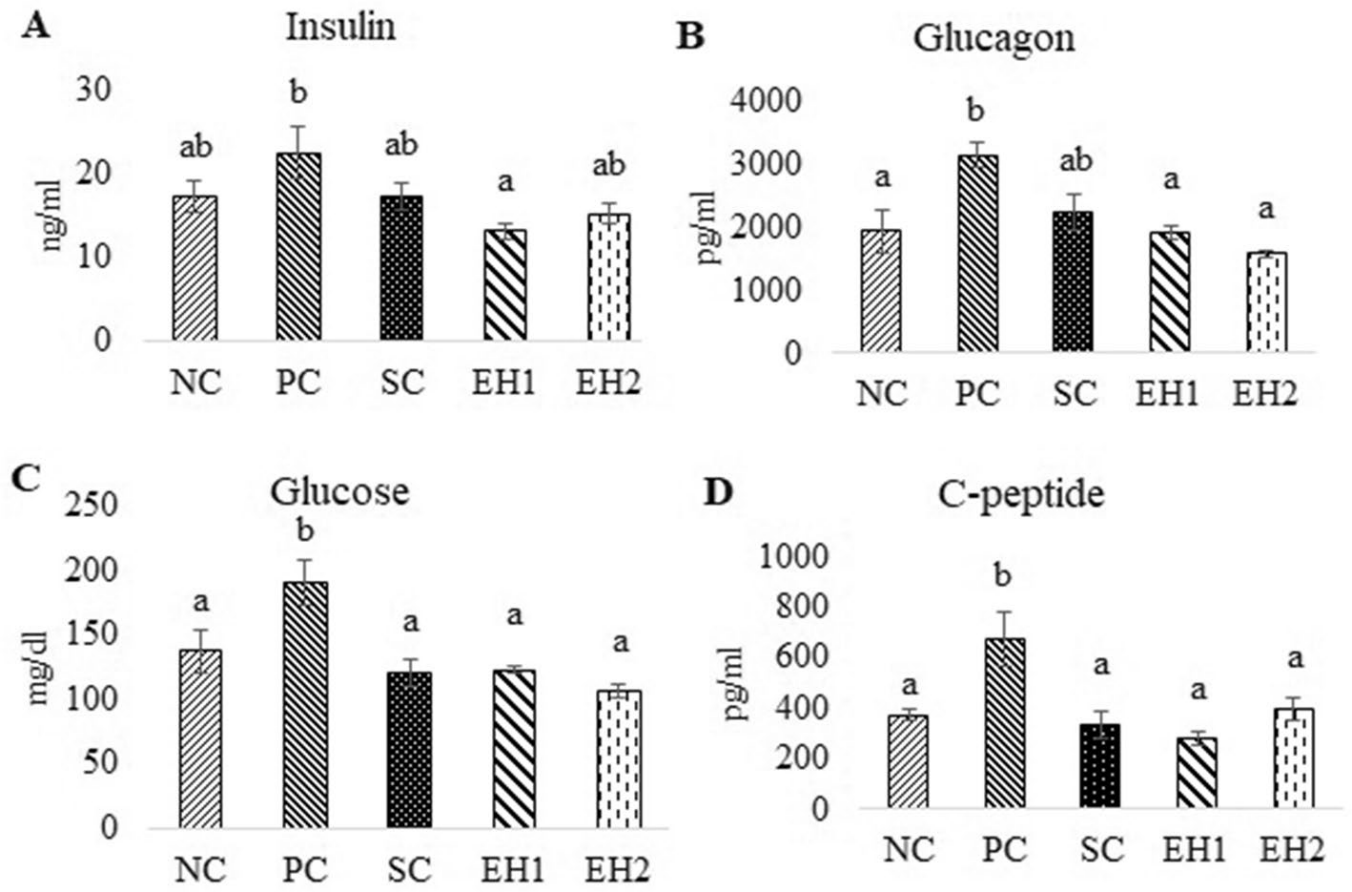
Effect of *Euphorbia helioscopia* on insulin, glucagon, glucose and C-peptide. Data are expressed as means ± standard errors of a total of 8 samples for each group. Bars with different letters are significantly different (*P* ≤ 0.05). NC, negative control group; PC, positive control group (Diabetic rats without any treatment); SC (Standard control group (Diabetic rats treated with metformin at 1 mg/kg body weight via oral lavage); EH1, Treatment group-I (Diabetic rats given methanolic extract of *Euphorbia helioscopia* at dose of 200 mg/Kg BW via oral lavage); EH2, Treatment group-II (Diabetic rats given methanolic extract of *Euphorbia helioscopia* at dose of 400 mg/Kg BW via oral lavage)

The serum level of insulin was decreased significantly (*P* ≤ 0.05) in the EH1 (13.08 ± 0.95^a^ ng/ml) group as compared to the PC (22.42 ± 3.03^b^ ng/ml) group. Decrease in serum insulin level was also observed in EH2 (15.16 ± 1.27^ab^ ng/ml), SC (17.23 ± 1.65^ab^ ng/ml), and NC groups (17.26 ± 1.84^ab^ ng/ml) as compared to the PC (22.42 ± 3.03^b^ ng/ml) group (Fig. 1: A) but the change was statistically non-significant. The mean serum levels of glucagon, glucose and C-peptide were significantly (*P* ≤ 0.05) increased in PC group (3135.78 ± 189.46^b^ pg/ml, 191.24 ± 17.75^b^ mg/dl, 671.70 ± 109.75^b^ pg/ml respectively) as compared to NC group (1928.58 ± 339.85^a^ pg/ml, 137.20 ± 16.49^a^ mg/dl, 372.85 ± 20.46^a^ pg/ml respectively) (Fig. 1: B, C, D). All these parameters were decreased significantly (*P* ≤ 0.05) in the EH1, EH2, and SC groups as compared to the PC group (Fig. 1: A, B, C, D).

#### Effect of *Euphorbia helioscopia* on carbohydrate metabolic enzymes

**Fig. 2:**
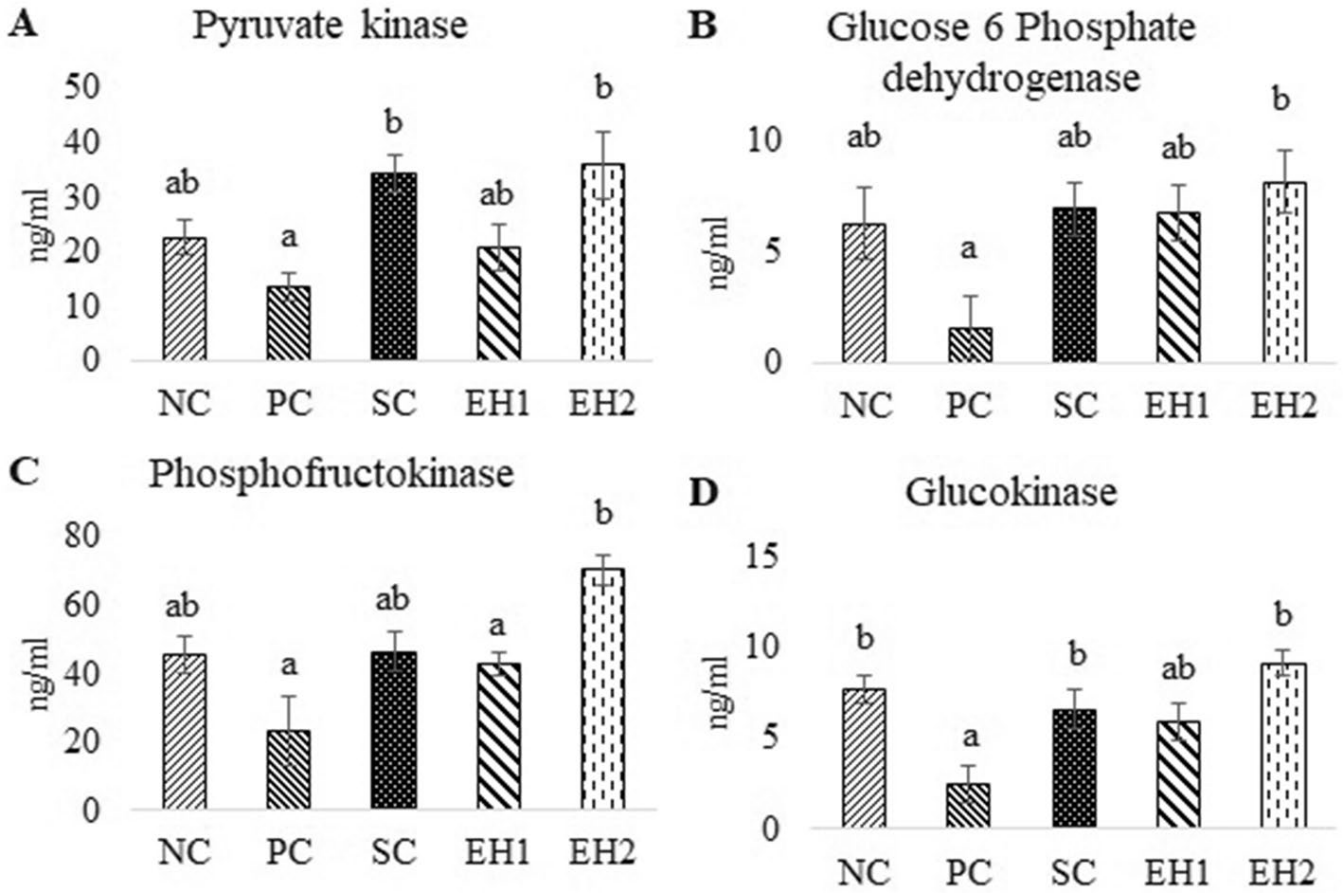
Effect of *Euphorbia helioscopia* on carbohydrate enzymes. Data are expressed as means ± standard errors of a total of 8 samples for each group. Bars with different lowercase letters are significantly different (*P* ≤ 0.05). NC, negative control group; PC, positive control group (Diabetic rats without any treatment); SC (Standard control group (Diabetic rats treated with metformin at 1 mg/kg body weight via oral lavage); EH1, Treatment group-I (Diabetic rats given methanolic extract of *Euphorbia helioscopia* at dose of 200 mg/Kg BW via oral lavage); EH2, Treatment group-II (Diabetic rats given methanolic extract of *Euphorbia helioscopia* at dose of 400 mg/Kg BW via oral lavage

Pyruvate Kinase, Glucose 6 phosphate dehydrogenase, phosphofructokinase, and glucokinase levels in serum were increased in the EH2 group (35.91 ± 6.19^b^ ng/ml, 8.12 ± 1.39^b^ ng/ml, 70.44 ± 4.4^b^ ng/ml, 9.1 ± 0.71^b^ ng/ml respectively) as compared to the PC group (13.69 ± 2.48^a^ ng/ml, 1.57 ± 1.43^a^ ng/ml, 23.29 ± 10.23^a^ ng/ml, 2.52 ± 1^a^ ng/ml respectively; Fig. 2: A, B, C, D). However, a significant difference was not seen in the serum levels of these enzymes in the EH1 group as compared to NC and SC groups.

#### Effect of *Euphorbia helioscopia* on amylin and leptin

Serum levels of amylin and leptin were significantly (*P* ≤ 0.05) decreased in the EH2 (2317.53 ± 63.15^b^ ng/ml and 0.67 ± 0.06^a^ ng/ml respectively), and SC (2075.1 ± 40.67^ab^ ng/ml and 1.425 ± 0.19^ab^ ng/ml respectively) groups in comparison to PC group (2962.45 ± 213.81^a^ ng/ml and 1.81 ± .39^b^ ng/ml respectively) but there was a non-significant decrease in the serum leptin levels in EH1 group when compared to the PC group. However, the decrease in serum amylin level of EH1 was significant as compared to the PC group (Table 2). The difference of both amylin and leptin in EH1 was non-significant from the EH2 group.

**Table 2:** Effect of *Euphorbia helioscopia* on Amylin, Leptin and Thyroid hormones

Groups	Amylin (pg/ml) ***p*** = 0.007	Leptin (ng/ml) ***p*** = 0.039	T3 (pg/ml) ***p*** = 0.019	T4 (ng/ml) ***p*** = 0.018	TSH (ng/ml) ***P*** = 0.03
**NC**	1736.35 ± 75.97^a^	1.06 ± 0.32^ab^	48.44 ± 0.47^ab^	59.97 ± 13.13^ab^	84.90 ± 21.38^ab^
**PC**	2962.45 ± 213.81^c^	1.81 ± .39^b^	40.66 ± 6.42^a^	18.94 ± 3.08^a^	51.42 ± 28.63^a^
**SC**	2075.1 ± 40.67^ab^	1.425 ± 0.19^ab^	54.97 ± 10.19^ab^	74.55 ± 14.94^b^	125.24 ± 11.71^b^
**EH1**	2078.19 ± 55.38^ab^	1.12 ± 0.05^ab^	70.35 ± 5.52^ab^	67.27 ± 9.38^ab^	117.03 ± 4.17^ab^
**EH2**	2317.53 ± 63.15^b^	0.67 ± 0.06^a^	77.35 ± 4.59^b^	73.67 ± 14.11^b^	123.09 ± 8.97^ab^

Data are expressed as means ± standard errors of a total of 8 samples for each group. Values having different lowercase letters in a column are significantly different (*P* ≤ 0.05). NC, negative control group; PC, positive control group (Diabetic rats without any treatment); SC (Standard control group (Diabetic rats treated with metformin at 1 mg/kg body weight via oral lavage); EH1, Treatment group-I (Diabetic rats given methanolic extract of *Euphorbia helioscopia* at dose of 200 mg/Kg BW via oral lavage); EH2, Treatment group-II (Diabetic rats given methanolic extract of *Euphorbia helioscopia* at dose of 400 mg/Kg BW via oral lavage; T3, triiodothyronine; T4, thyroxin, TSH; thyroid stimulating hormone

#### Effect of *Euphorbia helioscopia* on thyroid hormones

Mean values of T3 and T4 were significantly (*P* ≤ 0.05) increased in the EH2 group (77.35 ± 4.59^b^ pg/ml and 73.67 ± 14.11^b^ ng/ml respectively) as compared to the PC group (40.66 ± 6.42^a^ pg/ml and 18.94 ± 3.08^a^ ng/ml respectively). Statistically, a non-significant increase in T3 and T4 levels was also observed in the EH1 group compared to the PC group. Serum TSH level was also increased in the EH1 and EH2 groups as compared to the PC group in a dose-dependent manner. However, this increase in TSH was statistically non-significant. Supplementation of metformin also increased significantly (*P* ≤ 0.05) the serum TSH in the SC group as compared to the PC group (Table 2).

#### Effect of *Euphorbia helioscopia* on lipid profile

**Fig. 3:**
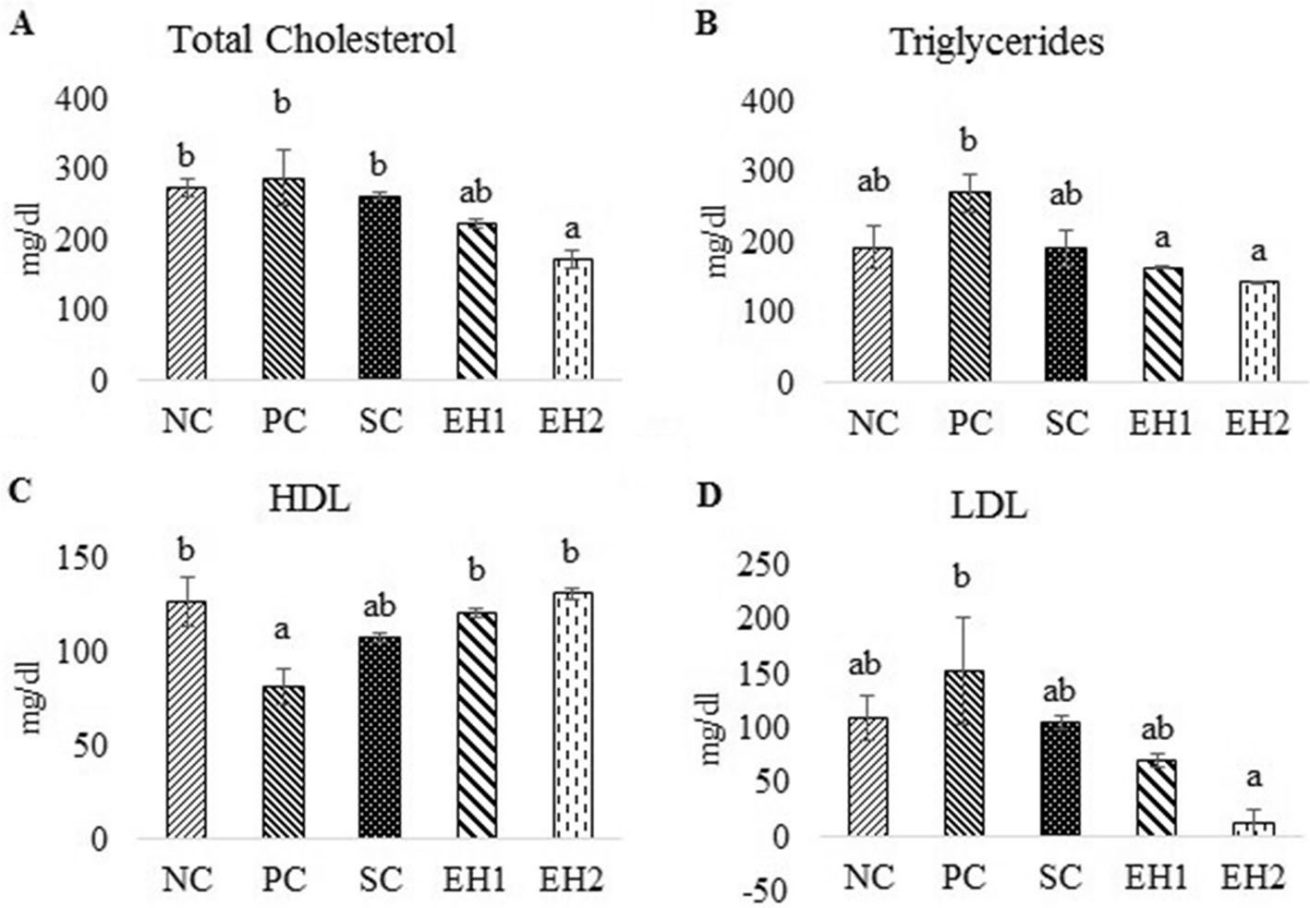
Effect of *Euphorbia helioscopia* on lipid profile. Data are expressed as means ± standard errors of a total of 8 samples for each group. Bars with different letters are significantly different (***P*** ≤ 0.05). NC, negative control group; PC, positive control group (Diabetic rats without any treatment); SC (Standard control group (Diabetic rats treated with metformin at 1 mg/kg body weight via oral lavage); EH1, Treatment group-I (Diabetic rats given methanolic extract of *Euphorbia helioscopia* at dose of 200 mg/Kg BW via oral lavage); EH2, Treatment group-II (Diabetic rats given methanolic extract of *Euphorbia helioscopia* at dose of 400 mg/Kg BW via oral lavage); HDL, high density lipoprotein; LDL, low density lipoprotein

Total cholesterol, LDL and triglyceride levels were significantly (*P* ≤ 0.05) decreased in EH2 group (143.27 ± 1.39^a^ mg/dl, 172.21 ± 12.41^a^ mg/dl, 12.46 ± 12.50^a^ mg/dl respectively) as compared to PC group (271.04 ± 27.39^b^ mg/dl, 288.17 ± 40.06^b^ mg/dl, 152.70 ± 49.86^b^ respectively) (Fig. 3: A, B, D). Whereas serum HDL levels were significantly increased in the EH1 (120.44 ± 2.41^b^ mg/dl) and EH2 groups (131.10 ± 2.79^b^ mg/dl) as compared to the PC group (81.27 ± 9.78^a^ mg/dl) and no significant change was observed among NC, EH1, and EH2 groups (Fig. 3: C). There was no significant difference in all the lipid profile parameters of the SC group as compared to the PC group.

#### Effect of *Euphorbia helioscopia* on pancreas histopathology

**Fig. 4:**
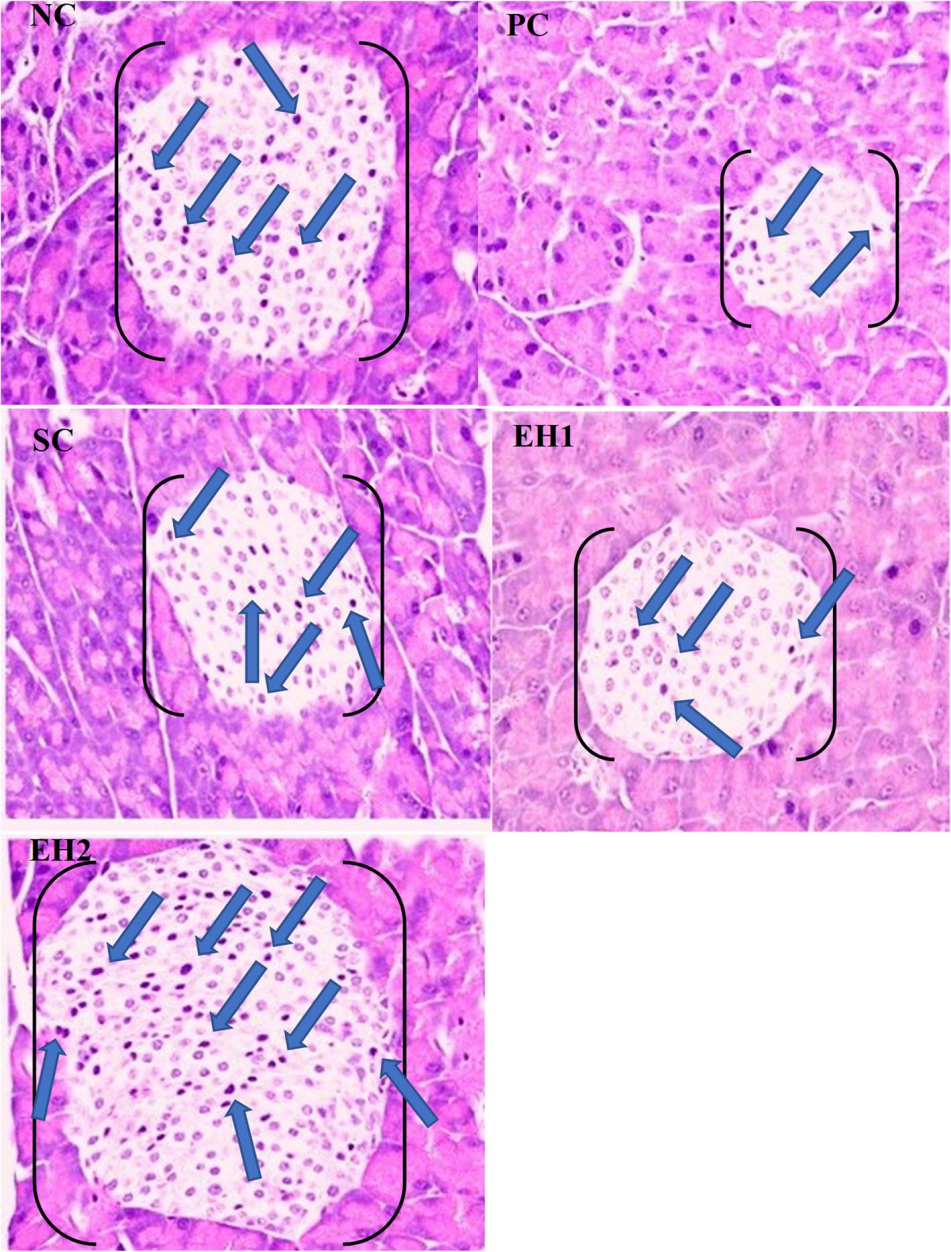
Effect of *Euphorbia helioscopia* on pancreas histopathology (H and E staining); 40X; NC: Photograph showing tissue of rat pancreas of negative control group; PC: Photograph showing tissue of rat pancreas of positive control group; SC: Photograph showing tissue of rat pancreas of standard control group; EH1: Photograph showing tissue of rat pancreas of methanolic extract of *Euphorbia helioscopia* at dose 200 mg kg; EH2: Photograph showing tissue of rat pancreas of methanolic extract of *Euphorbia helioscopia* at dose 400 mg kg. Brackets [] represent the size of islet of Langerhans and the blue colored arrows show the pancreatic beta cells

Tissue samples from the pancreas were collected from Wistar rats of each experimental group to analyze histopathological changes in pancreatic tissue. Photomicrographs showing histological changes in rat pancreas of all groups are presented in Fig. 4. As shown in Fig. 4. NC, the pancreas of rats in the negative control group had fully active islets of Langerhans with normal pancreatic beta cells marked with blue-colored arrows. However, in the PC group (diabetic rats), the pancreas showed destruction of β-cells, small-sized islets of Langerhans, and loss of cellular contents as shown in Fig. 4. PC. In the diabetic rats treated with MthEh at both doses, as shown in Fig. 4. EH1 and Fig. 4. EH2, there was a dose-dependent restoration of the normal histological structure showing normal pancreatic parenchyma with fully active β-cells in islets of Langerhans.

## Discussion

The present study was conducted to examine the potential of *Euphorbia helioscopia* (a medicinal plant) as an antioxidant and antidiabetic agent in a type 2 diabetic rat model. The impact of the *Euphorbia helioscopia* treatment on carbohydrate metabolism and thyroid gland was also evaluated. The antidiabetic and antioxidant effects of Euphorbiaceae family have been described previously in different animal models [28]. The rise in insulin and C-peptide levels in the untreated diabetic rats of the PC group is in contrast to type 2 diabetic animals, where elevated blood glucose is followed by increased insulin synthesis and insulin resistance resulting in hyperglycemia [29]. In humans with type 2 diabetes, the glucose challenge causes a substantially higher increase in blood glucose than in healthy individuals and is similar to that found in rats with diabetes caused by STZ-NA. Hyperglycemia observed after glucose load in type 2 diabetic humans normally results from both insulin resistance and impaired β-cell function [30–32], which is similar to our study where the insulin level is also increased along with glucagon and glucose in the PC group. But the treatment with methanolic extract of the plant significantly revered the levels of insulin, glucose, glucagon, and C-peptide in EH1 and EH2 groups compared with the metformin-treated group SC, which shows that the plant extract has reversed the β-cell function. This antidiabetic activity of the plant extract might be due to the antioxidant potential of this plant extract positively correlated with its antidiabetic potential in a previous in vitro study. This study explains that the methanolic extract of the plant is rich in chlorogenic acid, ferulic acid, caffeic acid, catechin, rutin, and quercetin. These phenolics and flavonoids are positively correlated with antioxidant and antidiabetic activity [15]. Previously it was observed that the antioxidant activity of the plant might be due to the presence of phenolic contents in its methanolic extract which are reported to play a vital role in scavenging the free radicals, preventing the oxidation process, and protecting cells from injury to avoid the danger of degenerative diseases including type II diabetes [15, 33–35]. The total antioxidant capacity was increased whereas the total oxidant status was decreased significantly in the current study. Moreover, the SOD, catalase, arylesterase, and paraoxonase activities were significantly increased whereas malondialdehyde levels were significantly decreased. Previous studies show that the methanolic extract of the plant shows antioxidant activity due to the presence of its phenolic contents [15, 21].

Due to the insufficiency of insulin, there is increased hepatic glucose output, failing to activate the enzymes for glycolysis and glycogenesis ultimately increasing blood glucose level [35]. Activities of glucokinase and glucose 6-phosphate dehydrogenase, phosphofructokinase, and pyruvate kinase were decreased remarkably in the positive control group in the current study. *Euphorbia helioscopia* and metformin-induced significant increase in serum glucose-6-phosphate dehydrogenase activity which might enhance the entry of glucose into the pentoses monophosphate shunt and this may cause an increase in producing the reducing agent, NADPH, with an associated reduction in oxidative stress [36]. The decline in pyruvate kinase activity observed in the current experiment suggests the decreased glucose utilization (glycolysis) and enhanced synthesis of glucose (gluconeogenesis) demonstrating that these pathways were changed [37]. *Euphorbia helioscopia* and metformin also enhanced the glucokinase and pyruvate kinase activity thereby inducing the active utilization of glucose. Carbohydrate metabolism and pancreatic hormones are effectively regulated by thyroid hormones [38]. In the current study, level of T3, T4 and TSH were decreased in the untreated diabetic group. The decrease in T3 may also be explained by impaired T4 conversion into T3 which is involved in the improvement of glycemic control [39]. Serum T3, T4 and TSH levels were restored after *Euphorbia helioscopia* treatment in current study. These findings are supported by the previous observations where a significant decrease in the level of serum thyroid hormone was observed in experimentally induced hyperglycemic rats [40]. The phenolic contents present in the methanolic extract are ultimately responsible for the correction of thyroid hormones by ameliorating the oxidative stress that leads to diabetes and thyroid hormone imbalance. Thyroid dysfunction results as a complication of diabetes, hence treating diabetes can restore the serum levels of thyroid hormones [41]. Leptin is a vital hormone derived from adipose tissue that has been observed to play a significant role in various pathways prompting the risk of diabetes [42]. A significant decrease in serum leptin levels observed after *Euphorbia helioscopia* treatment can be correlated with increased insulin sensitivity in the treated groups as previous studies also suggested an inverse relationship between insulin levels and leptin levels [43–45]. Amylin is a peptide hormone that is co-localized and co-secreted with insulin from β cells of the pancreas [21, 46, 47]. In the present study, a significant decrease in amylin levels was observed after *E. helioscopia* treatment. Total cholesterol, triglycerides, and LDL levels were reduced significantly whereas HDL levels were increased after *E. helioscopia* treatment. Previously it has been reported that methanolic extract of *E. helioscopia* lowered the total cholesterol, LDL, triglycerides and increased the HDL in the liver and serum of the paracetamol-induced oxidative stress of a mouse model [48]. *Euphorbia hirta* leaf extract treatment in diabetic rats decreased the elevated levels of TC, TG, LDL, and VLDL while HDL levels were increased to the normal values [49]. A possible mechanism in normalizing the lipid profile of diabetic rats treated with the plant extract may be the antioxidant activity of the plant’s phenolic contents that delay the lipid peroxidation process through their free radical scavenging activity [50]. In our previous report in which the methanolic extract was quantitatively analyzed for different phenolic contents including gallic acid, hydroxybenzoic acid, chlorogenic acid, caffeic acid, and vanillic acid, and flavonoid contents including catechin acid, quercetin and rutin showed maximum antioxidant ability by scavenging DPPH and ABTS radicals [15].

Histopathological investigation of the pancreas indicated that diabetic rats treated with methanolic extract of the plant in EH1 and EH2 groups resulted in substantially normalizing the histoarchitecture of the endocrine portion of the pancreas particularly the β-cells in islets of Langerhans in a dose-dependent manner. An increase in the number of insulin-producing β-cells in pancreatic islets of Langerhans confirmed that a phenomenon of regeneration was activated in plant extracts treated groups. Diabetic rats of the PC group showed a significant reduction in the size of islets of Langerhans, pancreatic β-cells destruction, loss of cellular contents, nuclear shrinkage, and pyknosis, indicating the necrotic changes. These degenerative effects exerted by streptozotocin on pancreatic β-cells could be explained by its potential to generate reactive oxygen species (ROS) inside the β-cells and over-production of insulin from the pancreatic beta cells to compensate for insulin insufficiency due to insulin resistance [51]. The generation of these ROS leads to simultaneous lipid peroxidation and a massive rise in the concentration of cytosolic calcium resulting in rapid destruction of insulin-secreting pancreatic β-cells [52]. Moreover, streptozotocin also enhanced the β-cells damage by decreasing the activities of antioxidant enzymes inside the pancreas [52]. Treatment with the plant extract enhanced the viability, proliferation, and regeneration of destructed β-cells possibly by their protective and antioxidant potential leading to the subsequent increase in insulin production and secretion. These results are supported by the previous research studies [53]. This improvement in the beta cells by *E. helioscopia* methanolic extracts treatments in the same line with many studies which have reported that quercetin in the methanolic extract of *E. helioscopia* can inhibit pancreatic damage, supporting the regeneration of the pancreatic islets and enhancing its power to keep normal blood glucose levels in diabetes-induced rats [53, 54].

The main focus of the current research was to evaluate the effect of methanolic extract of *E. helioscopia* on the carbohydrate metabolic enzymes, oxidative stress, and antidiabetic activity. However, the study was limited to the evaluation of these parameters in the serum of the diabetic model of male rats evaluating the effect of the plant extract on the pancreas tissues. Moreover, the components of the plant extract were not separated, to examine the effect of plant extract as a whole in the initial stage of the research. The female rats were not included in the study due to the protective effect of female hormones, such as estrogen, against metabolic abnormalities [55]. As a future perspective, the current study will be extended to evaluate the toxic and therapeutic effects of each bioactive component of the plant extract on the antidiabetic activity, oxidative stress, and carbohydrate metabolism at a molecular and genetic level.

## Conclusion

In conclusion oral treatment with *Euphorbia helioscopia* methanolic extract was able to modulate the carbohydrate metabolic enzymes Pyruvate Kinase, Glucose 6 phosphate dehydrogenase, phosphofructokinase, and glucokinase in serum and can mitigate the oxidative stress and improve the activity of the antioxidant enzymes ultimately reversing the pathogenesis of type 2 diabetes. According to the results of current study the selected plant is recommended for its antidiabetic use. However, its bioactive constituents having the anti-inflammatory and antioxidant activity must be characterized and isolated for further investigation of its molecular mechanism involved at cellular level. So, as a future perspective they can be used as antidiabetic drug agent in human population.

## Data Availability

The datasets used and/or analyzed during the current study are available from the corresponding author on reasonable request.

## Abbreviations

TOS: Total Oxidant Status
MDA: Malondialdehyde
TBARS: Thiobarbituric reactive substance assay
TBA: Thiobarbituric acid
TAC: Total antioxidant capacity
NC: Negative control group
PC: Positive control group (Diabetic rats without any treatment)
SC: Standard control group (Diabetic rats treated with metformin at 1 mg/kg body weight via oral lavage)
EH1: Treatment group-I (Diabetic rats given methanolic extract of *Euphorbia helioscopia* at dose of 200 mg/Kg BW via oral gavage)
EH2: Treatment group-II (Diabetic rats given methanolic extract of *Euphorbia helioscopia* at dose of 400 mg/Kg BW via oral gavage)
